# Factors associated with one-night stands among men who have sex with men recruited via the internet: a cross-sectional survey in China

**DOI:** 10.3389/fpubh.2026.1781863

**Published:** 2026-03-17

**Authors:** Zhongrong Yang, Jicun You, Zhenguo Zhu, Jiasheng Qin, Guojun Jiang, Jie Dai, Hongyan Wang, Ming Gan, Xiuxiu Sun, Weiyong Chen, Feilin Ren, Jianyong Shen

**Affiliations:** 1 Department of AIDS/TB Control and Prevention, Huzhou Center for Disease Control and Prevention, Huzhou, Zhejiang, China; 2 Department of AIDS/TB Control and Prevention, Anji Center for Disease Control and Prevention, Huzhou, Zhejiang, China; 3 Department of AIDS/TB Control and Prevention, Depqing Center for Disease Control and Prevention, Huzhou, Zhejiang, China; 4 Department of AIDS/TB Control and Prevention, Changxing Center for Disease Control and Prevention, Huzhou, Zhejiang, China; 5 Department of AIDS/TB Control and Prevention, Nanxun Center for Disease Control and Prevention, Huzhou, Zhejiang, China; 6 Department of AIDS/TB Control and Prevention, Wuxing Center for Disease Control and Prevention, Huzhou, Zhejiang, China; 7 Department of HIV/STD Control and Prevention, Zhejiang Provincial Center for Disease Control and Prevention, Hangzhou, Zhejiang, China; 8 Zhejiang Key Lab of Vaccine, Infectious Disease Prevention and Control, Hangzhou, Zhejiang, China

**Keywords:** associated factors, HIV, internet, men who have sex with men, one-night stands, risk

## Abstract

**Objective:**

One-night stands among men who have sex with men (MSM) significantly increase the risk of HIV transmission. Given the growing role of the Internet in facilitating such encounters, this study examined the factors associated with one-night stands among Internet-recruited MSM in China to identify actionable targets for behavioral interventions and HIV prevention strategies.

**Methods:**

The study participants were MSM recruited online in May 2024 through a non-governmental organization, and a questionnaire survey was conducted. The participants were divided into two groups based on whether they had experienced one-night stands or not (i.e., one-night stand and non-one-night stand groups). Univariate and multivariate logistic regression analyses were employed to analyze the factors associated with the occurrence of one-night stands among the participants.

**Results:**

A total of 604 participants were surveyed, 270 of whom reported having one-night stands and accounted for 44.70% (270/604). The results of multivariate logistic regression analysis showed that older age groups, meeting homosexual partners in offline fixed venues (such as bars, karaoke television, and saunas), seeking male partners primarily via the Internet/dating apps, having anal intercourse with homosexual partners in the last 6 months, engaging in behaviors such as drinking, drug use (including new drugs), or using aphrodisiacs (such as Viagra) during sexual encounters, and believing that condoms can effectively prevent HIV transmission were associated with a higher likelihood of having one-night stands. Conversely, participants who had a regular homosexual partner or knew their homosexual partner's HIV status had a lower likelihood of experiencing one-night stands.

**Conclusion:**

The proportion of participants who had one-night stands was relatively high, indicating the need for increased public education within the MSM population. It is essential to promote consistent condom use during anal intercourse, maintain regular homosexual partners, and enhance HIV testing coverage. Furthermore, efforts should be made to avoid behaviors such as drinking, drug use, and using aphrodisiacs during sexual encounters to reduce the risk of HIV and other sexually transmitted infections in MSM.

## 1 Introduction

The HIV epidemic continues to be one of the foremost public health challenges worldwide (1, 2). Achieving the UNAIDS 95–95–95 targets necessitates effective testing and the diagnosis of individuals infected with HIV (3). The widespread availability of antiretroviral therapy has enabled many individuals living with HIV to manage the virus effectively, mitigate transmission risks, and enhance their quality of life (4). However, despite significant advancements in medical technology, numerous regions still encounter obstacles such as inadequate treatment resources, social stigma, and insufficient comprehensive sex education, which contribute to the persistently high rates of HIV transmission (5). In recent years, governments have implemented a range of measures to combat AIDS, including interventions that target high-risk behaviors, free testing, antiviral treatment, and health education. Nonetheless, the stigma associated with HIV and existing biases against high-risk populations, such as men who have sex with men (MSM), continue to hinder efforts to address the AIDS epidemic (6, 7).

In recent years, the MSM community has garnered increasing global attention, particularly regarding sexual health and social relationships (8–10). The sexual behavioral patterns of MSM are influenced by various factors (11), among which one-night stands represent a significant behavioral expression (12). One-night stands are characterized by anonymity and temporality, with many MSM seeking brief sexual satisfaction through digital platforms (13). This rapid form of interaction often occurs on social media applications and dating websites, providing participants with the flexibility to choose. The MSM community is more open to exploring their sexual needs and preferences. One-night stands facilitate new sexual experiences, fulfill physiological needs, and provide personal freedom; sexual behavior encompasses not only physical satisfaction but also emotional release and affirmation of sexual identity (14). In some instances, one-night stands not only fulfill physiological needs but also constitute social activities, as participants may meet new partners through gatherings or events, thereby enhancing social interactions (15). However, these behaviors may carry significant health risks, especially in the absence of awareness of safe sexual practices, thereby increasing the risk of HIV and other sexually transmitted infections (STIs).

In recent years, rapid and convenient means of communication, facilitated by digital social platforms and the Internet, have simplified the process of finding casual hookups (16). Many MSM seek instant gratification through mobile applications, a trend that has contributed to the growing popularity of one-night stands to some extent. Within the MSM community, the phenomenon of one-night stands is characterized by various attributes and deep-rooted influencing factors. Understanding these characteristics and their underlying causes is essential to promote effective health interventions, encourage positive sexual behaviors, and establish healthy social relationships. Currently, there is a paucity of research on the correlates of one-night stands among MSM recruited via the Internet. This study aimed to address this gap by exploring the correlates associated with the characteristics of the MSM population and analyzing their social interaction patterns and sexual behavior traits. Specifically, this research was designed to move beyond identifying risk factors to determining which of these factors are most amenable to change through public health interventions. By clarifying the pathways linking Internet use, substance use, and partnership status to one-night stands, this cross-sectional study provides empirical evidence to guide the refinement of HIV prevention messages, the design of situational risk reduction tools, and the allocation of resources toward the most impactful intervention points within the digital MSM community.

## 2 Materials and Methods

### 2.1 Study design and ethical statement

This study was a cross-sectional survey conducted through online recruitment of MSM in May 2024 by a non-governmental organization, utilizing the Internet for a questionnaire survey (17). The research protocol was approved by the Ethics Committee of the Huzhou Center for Disease Control and Prevention (Approval No: HZ2023003). Only participants who provided informed consent were included in the study.

### 2.2 Participants

This study utilized Internet recruitment rather than random sampling, thereby employing a nonprobability sampling method similar to snowball sampling. The following calculation method was used to determine the sample size: Based on behavior monitoring data obtained from repeated surveys, we derived the formula N = 400 × Q/P. Here, P represents the estimated proportion of relevant behaviors occurring during the survey period, which is based on the reported proportion of MSM engaging in unprotected anal intercourse, which is estimated to be between 28.7% and 53.0% according to relevant surveys (18–20). In this study, we used *P* = 47.5% and *Q* = 1–P. Considering these parameters, the study required the recruitment of at least 442 participants via the Internet.

The participants were MSM recruited online. The Wenjuanxing platform was configured so that each WeChat account could submit the questionnaire only once. Quality control measures were implemented through various restrictions on questionnaire completion, such as mandatory questions, logical skips, and limitations on responses to ensure that the questionnaires met the recruitment criteria. The investigators underwent standardized training and used a uniform questionnaire to complete the survey. Before the survey, the investigators explained the purpose, significance, methodology, and privacy protection policies to participants, including this information at the beginning of the questionnaire. Participants were informed that the purpose of the survey was to develop prevention strategies for HIV and STIs among MSM, that the survey was anonymous, and that only aggregate data would be analyzed without any individual data being considered. A total of 626 participants completed the online questionnaire, of which 604 provided informed consent and completed the questionnaire; 22 individuals who declined to participate were excluded.

The inclusion criteria were individuals aged ≥18 years, who had engaged in sexual activities with males (including masturbation, oral sex, and anal sex), and who were willing to participate in the survey after providing informed consent. We excluded participants aged < 18 years, those who had sexual partners recruited offline or at fixed locations, those who were unwilling to participate after providing informed consent, and those who self-reported having mental or cognitive disorders.

### 2.3 Survey content

Our questionnaire design was based primarily on a sentinel surveillance questionnaire for MSM in China and a survey questionnaire for college students (21–23). After conducting a pilot survey and improving the variable settings of the questionnaire, the formal survey was conducted. The main contents of the survey included general sociodemographic characteristics (age, marital status, place of residence, education level, and average monthly income), characteristics related to sexual behavior (sexual attitudes, condom use during sexual activities, whether currently having a regular sexual partner, whether sexual activity occurred in the past 6 months, and alcohol or drug use during homosexual activity), whether HIV prevention services were received in the past year, whether HIV testing was conducted in the past year, perceived self-efficacy regarding condom use, and knowledge of the HIV infection status of homosexual partners, among other information (17).

### 2.4 Definition of relevant indicators

#### 2.4.1 One-night stands

This refers to casual sexual encounters between MSM, characterized by a single sexual relationship that occurs voluntarily based on physiological impulses, sensory attraction, or immediate needs among MSM who do not know each other, without emotional commitment, follow-up contact, or transactions involving money or goods.

#### 2.4.2 Commercial sexual activities

These refer to sexual encounters that involve financial transactions, such as prostitution.

#### 2.4.3 HIV prevention services

This refers to whether individuals have received AIDS-related promotional services such as free condoms, counseling, and training in the past year.

#### 2.4.4 Self-efficacy in condom use

This was measured using the scale developed by Hanna et al. (24) to assess self-efficacy in condom usage. It primarily included three questions: (1) Before engaging in sexual activity, do you feel confident discussing condom use with your sexual partner? (2) If your partner refuses to use a condom or does not have one, do you feel unconfident about not engaging in sexual activity? (3) Before engaging in sexual activity, do you feel confident about preparing your condoms in advance? Each of the three questions had five response options: very confident, quite confident, somewhat confident, not confident, and very unconfident, with scores of 3, 2, 1, 0, and −1, respectively. The total scores were categorized into three groups: 9 points, 5–8 points, and < 4 points. The Cronbach's alpha coefficient for this measurement was 0.816.

### 2.5 Statistical analysis

Statistical analyses were performed using R version 4.4.1. Continuous data are presented as mean ± standard deviation. Categorical data are expressed as counts or percentages using the chi-square test. Participants were divided into two groups based on whether they engaged in one-night stands with their homosexual partners (1 = yes, 0 = no). Variables with a *P*-value < 0.2 from the univariate regression analysis were included as independent variables in the model, followed by multivariate logistic regression analysis using the Enter method. Statistical significance was set at *P* < 0.05.

## 3 Results

### 3.1 General demographic characteristics

A total of 604 participants were recruited for this study, with a minimum age of 19 years and a maximum age of 53 years, resulting in an average age of (28.04 ± 6.43) years. Participants aged 19–25 years accounted for 40.23% (243/604). Those who were unmarried or divorced comprised 93.05% (562/604). Rural residents accounted for 38.58% of the participants (233/604). Those with an average monthly income of 6,000 CNY or less represented 58.61% of the participants (354/604). Individuals with a high school education or lower constituted 22.52% of the participants (136/604), and students accounted for 20.53% (124/604). Table 1 presents the results of the study.

**Table 1:** General demographic characteristics of participants.

**Variables**	**Whether the participant had a one-night stand with a homosexual partner**				***x^2^***	***P***
	**Yes (*****n*** = **270)**		**No(*****n*** = **334)**			
	***n***	***%***	***n***	***%***		
**Age (yrs)**						
19–25	85	35.0	158	65.0	14.896	< 0.001^**^
26–53	185	51.2	176	48.8		
**Marital status**						
Married or cohabiting	23	54.8	19	45.2	1.437	0.231
Unmarried or divorced	247	44.0	315	56.0		
**Hometown of origin**						
Urban area	169	45.6	202	54.4	0.199	0.655
Rural area	101	43.3	132	56.7		
**Educational level**						
High school or below	62	45.6	74	54.4	0.019	0.890
College degree or above	208	44.4	260	55.6		
**Student**						
No	227	47.3	253	52.7	5.843	0.016^*^
Yes	43	34.7	81	65.3		
**Monthly living expenses (CNY** ^#^ **)**						
≤ 6000	147	41.5	207	58.5	3.188	0.074
>6000	123	49.2	127	50.8		

^#^CNY, Chinese Yuan.

^*^P < 0.05.

^**^P < 0.01.

### 3.2 Analysis of factors associated with one-night stands among participants

In this study, 270 participants (44.70 %) had one-night stands with homosexual partners. The results of univariate analysis (Tables 1, 2) showed that variables with *P* < 0.2 included age, current student status, average monthly income, whether the participant believes that using party drugs such as Rush Popper increases the risk of HIV infection, whether they have learned about HIV information through media in the past year, whether they have received HIV prevention services in the past year, whether they currently have a steady homosexual partner, whether they met their homosexual partner through offline fixed venues (such as bars, karaoke televisions, and saunas), whether their primary method of finding male partners is the Internet/dating apps, whether they have engaged in anal sex with homosexual partners in the last 6 months, whether they have consumed alcohol, used drugs or taken enhancement drugs during sexual activities with homosexual partners, whether they have had sexual encounters with opposite-sex partners in the last 6 months, whether they are aware of their homosexual partner's HIV status, whether they believe condoms can effectively prevent HIV transmission among homosexual individuals, whether they have been tested for HIV in the past year, and whether they have used post-exposure prophylaxis (PEP) in the last 6 months.

**Table 2:** Analysis of factors associated with one-night stands among participants.

**Variables**	**Whether the participant had a one–night stand with a homosexual partner**				**Univariate analysis**		**Multivariate analysis**	
	**Yes (*****n*** = **270)**		**No (*****n*** = **334)**		**OR (95%CI)**	**P**	**OR (95%CI)**	**P**
	***n***	***%***	***n***	***%***
**Age (yrs)**								
19–25	85	35.0	158	65.0	Ref.		Ref.	
26–53	185	51.2	176	48.8	1.95 (1.40–1.32)	< 0.001^**^	1.73 (1.12–2.70)	0.014^*^
**Marital status**								
Married or cohabiting	23	54.8	19	45.2	Ref.		Ref.	
Unmarried or divorced	247	44.0	315	56.0	0.65 (0.34–1.21)	0.177	0.83 (0.37–1.83)	0.637
**Student**								
No	227	47.3	253	52.7	Ref.		Ref.	
Yes	43	34.7	81	65.3	0.59 (0.39–0.89)	0.012^*^	0.79 (0.45–1.38)	0.409
**Monthly living expenses (CNY** ^#^ **)**								
≤ 6000	147	41.5	207	58.5	Ref.		Ref.	
>6000	123	49.2	127	50.8	1.36 (0.98–1.89)	0.062	1.12 (0.75–1.68)	0.577
**Whether you think using enhancers like Rush Popper increases the risk of HIV infection**								
No	76	40.4	112	59.6	Ref.		Ref.	
Yes	194	46.6	222	53.4	1.29 (0.91–1.83)	0.156	1.09 (0.72–1.65)	0.676
**Whether AIDS related information has been learned through online media in the past year**								
No	23	33.3	46	66.7	Ref.		Ref.	
Yes	247	46.2	288	53.8	1.72 (1.02–2.95)	0.045^*^	1.22 (0.64–2.33)	0.550
**Whether you received HIV/AIDS prevention services in the past year**								
No	73	36.5	127	63.5	Ref.		Ref.	
Yes	197	48.8	207	51.2	1.66 (1.17–2.35)	0.004^**^	1.47 (0.94–2.29)	0.089
**Whether you have a stable homosexual partner**								
No	186	47.3	207	52.7	Ref.		Ref.	
Yes	84	39.8	127	60.2	0.74 (0.52–1.03)	0.077	0.42 (0.27–0.63)	< 0.001^**^
**Whether met homosexual partners through fixed offline venues**								
No	188	39.9	283	60.1	Ref.		Ref.	
Yes	82	61.7	51	38.3	2.42 (1.64–3.61)	< 0.001^**^	1.61 (1.02–2.56)	0.043^*^
**Whether the main place/way to find homosexual partners is the Internet/dating software**								
No	29	32.2	61	67.8	Ref.		Ref.	
Yes	241	46.9	273	53.1	1.86 (1.16–3.02)	0.011^*^	2.04 (1.16–3.68)	0.015^*^
**Whether you had anal sex with homosexual partners in the past six months**								
No	72	29.0	176	71.0	Ref.		Ref.	
Yes	198	55.6	158	44.4	3.06 (2.18–4.34)	< 0.001^**^	3.34 (2.23–5.06)	< 0.001^**^
**Whether you have been engaged in alcohol consumption, drug use, or the use of sexual enhancement drugs (such as Viagra) when engaging in sexual activity with**								
**homosexual partners**								
No	205	39.6	313	60.4	Ref.		Ref.	
Yes	65	75.6	21	24.4	4.73 (2.85–8.14)	< 0.001^**^	4.89 (2.76–8.95)	< 0.001^**^
**Whether you have been engaged in sexual activity with heterosexual partners in the past six months**								
No	233	42.9	310	57.1	Ref.		Ref.	
Yes	37	60.7	24	39.3	2.05 (1.20–3.56)	0.009^**^	1.47 (0.75–2.89)	0.261
**Condom use self–efficacy measurement (Scores)**								
4 or below	79	45.4	95	54.6	Ref.			
5–8	97	49.7	98	50.3	1.19 (0.79–1.79)	0.405		
9	94	40.0	141	60.0	0.80 (0.54–1.19)	0.275		
**Whether you want to know the HIV infection status of your homosexual partner**								
No	27	40.3	40	59.7	Ref.			
Yes	243	45.3	294	54.7	1.22 (0.73–2.07)	0.443		
**Whether you are aware of the HIV infection status of your homosexual partner**								
No	95	49.5	97	50.5	Ref.		Ref.	
Yes	175	42.5	237	57.5	0.75 (0.53–1.06)	0.107	0.62 (0.41–0.95)	0.027^*^
**Whether you are worried about getting HIV from your homosexual partner**								
No	91	43.3	119	56.7	Ref.			
Yes	179	45.4	215	54.6	1.09 (0.78–1.53)	0.621		
**Whether you believe that condoms can effectively prevent the transmission of HIV during homosexual encounters**								
No	19	25.0	57	75.0	Ref.		Ref.	
Yes	251	47.5	277	52.5	2.72 (1.60–4.81)	< 0.001^**^	2.95 (1.55–5.84)	0.001^**^
**Whether you have been tested for HIV in the past year**								
No	35	29.4	84	70.6	Ref.		Ref.	
Yes	235	48.5	250	51.5	2.26 (1.48–3.51)	< 0.001^**^	1.13 (0.66–1.93)	0.663
**Whether you have used HIV post exposure prophylaxis drugs in the past six months**								
No	244	43.4	318	56.6	Ref.		Ref.	
Yes	26	61.9	16	38.1	2.12 (1.12–4.11)	0.023^*^	1.36 (0.62–3.05)	0.447

^#^CNY, Chinese Yuan.

^*^P < 0.05.

^**^P < 0.01.

**Figure 1:**
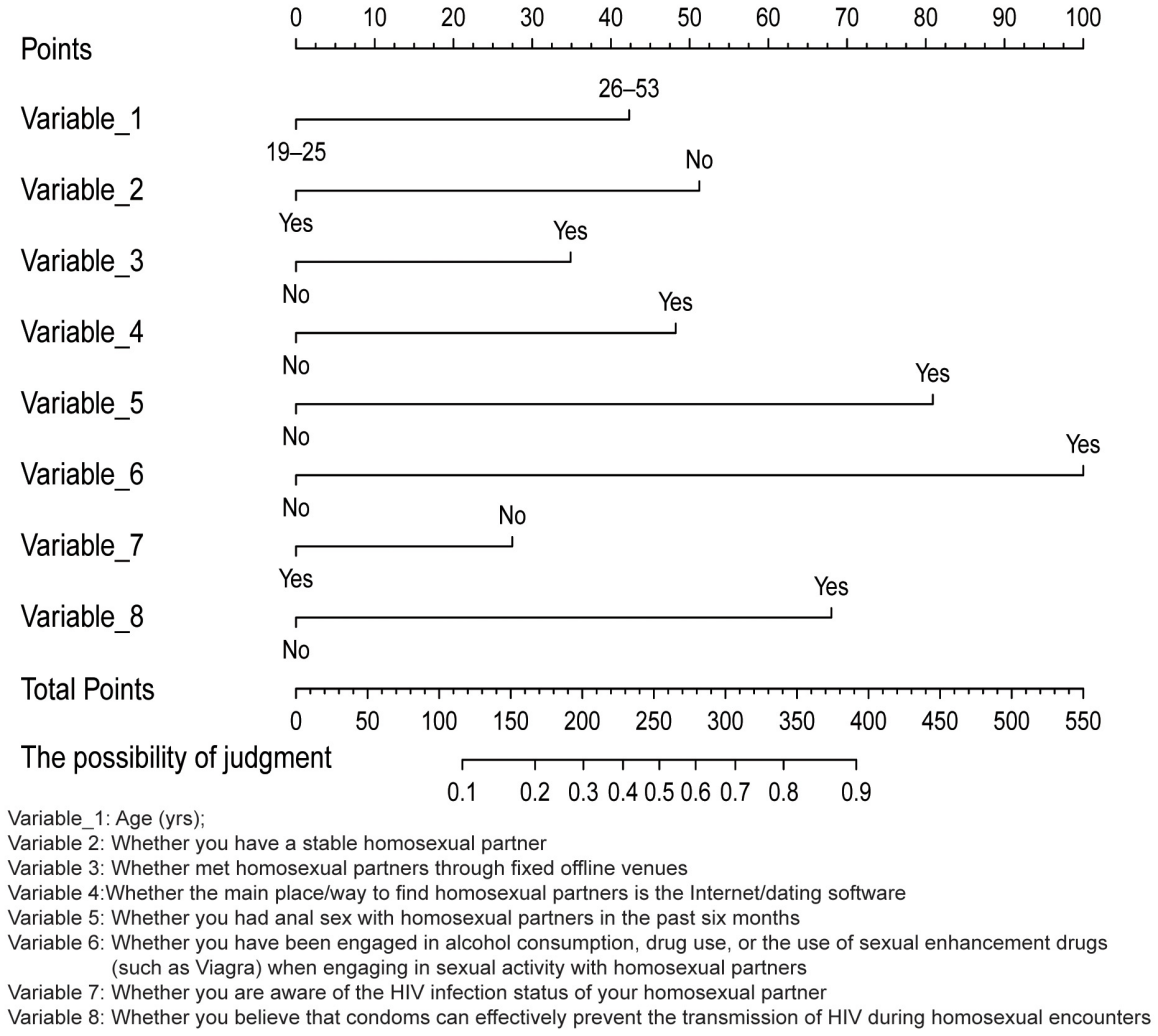
A nomogram of factors associated with one-night stands for the possibility of judgment.

Incorporating these variables into a multivariate logistic regression analysis, the results (Table 2) indicated that the likelihood of participants aged 26–53 engaging in one-night stands increases by 73% [adjusted odds ratio (aOR) = 1.73; 95% confidence interval (CI) = 1.12–2.70]. Participants who met their homosexual partners through offline fixed venues (such as bars, karaoke televisions, and saunas) showed a 61% increase in the likelihood of engaging in one-night stands (aOR = 1.61; 95% CI = 1.02–2.56). Those whose primary method of finding male partners is the Internet/dating apps showed a 104% increased likelihood of having one-night stands (aOR = 2.04; 95% CI = 1.16–3.68). Participants who engaged in anal sex with homosexual partners in the last 6 months showed a 234% increased likelihood of having one-night stands (aOR = 3.34; 95% CI = 2.23–5.06). The likelihood of having one-night stands increased by 389% (aOR = 4.89; 95% CI = 2.76–8.95) among participants who had consumed alcohol, used drugs, or engaged in enhancement drug use during sexual activities with homosexual partners. Participants who believe that condoms can effectively prevent HIV transmission among homosexual individuals showed a 195% increased likelihood of engaging in one-night stands (aOR = 2.95; 95% CI = 1.55–5.84). Conversely, participants who had a steady homosexual partner showed a 58% lower likelihood of engaging in one-night stands (aOR = 0.42; 95% CI = 0.27–0.63). Additionally, participants who were aware of their homosexual partners' HIV status showed a 38% lower likelihood of engaging in one-night stands (aOR = 0.62; 95% CI = 0.41–0.95). Figure 1 shows the associated factor analysis whether the participants engaged in one-night stands.

## 4 Discussion

In this study, we performed an in-depth analysis of the factors influencing whether MSM recruited via the Internet engage in one-night stands. Traditionally, face-to-face social methods and venues such as bars and clubs have served as the primary channels for friendship formation and sexual encounters. However, the rise of digital social platforms is transforming patterns of social interaction among MSM (25). These platforms enhance accessibility and anonymity, allowing MSM to communicate and interact more conveniently and efficiently (26, 27). Users can engage in conversations without disclosing their true identities, which alleviates the social pressures and discrimination associated with their sexual orientation (5). In this environment, MSM can express their sexual needs, seek emotional support, and establish relationships without fear of judgment from others. This study not only examined the behavioral patterns of MSM but also analyzed their coping strategies in response to health risks. This study addresses the existing gaps in the literature and provides empirical support for the development of relevant health interventions.

### 4.1 Age

The results of this study indicate that, compared to younger individuals aged 25 years and below, MSM in the age group of 26–53 years are 73% more likely to engage in one-night stands. This finding warrants a deeper examination of the differences in sexual behavior patterns across various age groups.

Age is typically correlated with increased personal life experience and psychological maturity. For men aged 26–53 years, greater career maturity, economic stability, and emotional resilience may lead to enhanced confidence in seeking sexual partners. In contrast to younger individuals, men in this age group may be more inclined to adopt a casual perspective toward sexual relationships, opting for one-night stands to satisfy physiological needs, rather than seeking deeper emotional connections. Sociocultural changes may have significantly influenced these findings. In recent years, increased societal recognition and acceptance of the MSM community have led to the proliferation of social platforms and dating applications, thus increasing the opportunities and frequency of one-night stands. However, for MSM, such encounters are often associated with a higher risk of HIV and other STIs. Therefore, providing appropriate safe sex education and HIV testing resources for MSM aged 26–53 years could effectively mitigate the risk of STIs.

### 4.2 Offline fixed venues

Fixed venues, such as bars, karaoke televisions, and bathhouses, frequently serve as gathering places for MSM, offering a relatively open and inclusive social environment (28). The findings of this study indicated that meeting homosexual sexual partners at these venues increased the likelihood of engaging in one-night stands by 61%.

Atmospheres characterized by noise and intoxication often foster the occurrence of one-night stands, prompting men to partake in non-committal sexual behaviors driven by fleeting impulsive urges. Psychologically, many MSM experiencing emotional or social loneliness seek brief relationships in these environments to alleviate their inner emptiness. Offline direct contact can provide immediate gratification; however, such relationships often lack depth and stability. However, this phenomenon poses potential health risks. Partners encountered in establishments such as bars and karaoke televisions are often insufficiently familiar with each other, significantly increasing the risk of STIs. Therefore, it is crucial to implement more rigorous and targeted education and awareness campaigns to promote safe sexual practices and regular testing.

### 4.3 Internet and dating applications

In contemporary society, the proliferation of the Internet and dating applications has provided MSM with novel means of social interaction (29, 30). The findings of this study indicate that MSM who seek male sexual partners primarily through the Internet and dating apps have a 104% increased likelihood of engaging in one-night stands.

These platforms offer a more convenient and efficient method for finding partners, allowing users to browse potential matches in a comfortable environment and swiftly establishes connections. This immediacy alleviates the nervousness and uncertainty often associated with traditional social interactions, facilitating individuals' expression of their needs and desires, and thus promoting one-night stands. Furthermore, the anonymity and privacy protection afforded by these applications permit MSM to feel more at ease when seeking sexual partners. Compared with face-to-face interactions, online communication effectively mitigates social pressure and judgment, enabling individuals who were previously reluctant to disclose their sexual orientation to explore more open relationships. However, this trend presents significant safety and health challenges. In the online environment, the absence of face-to-face contact often results in dating app users displaying weaker emotional investment and a diminished sense of responsibility. The short-term nature of one-night stands may exacerbate health risks; therefore, health education and the promotion of safe sexual practice within MSM communities are particularly vital. Enhancing user risk awareness and providing health resources through online platforms, such as information on condom use and access to pre- and post-exposure prophylaxis for HIV, are crucial measures for mitigating the risks associated with one-night stands.

### 4.4 Anal intercourse

Anal intercourse, as a specific form of sexual behavior, is typically associated with higher levels of emotional and physical intimacy (31). The findings of this study indicate that among MSM who have engaged in anal intercourse in the past 6 months, the likelihood of experiencing a one-night stand increases by 234%.

After engaging in anal intercourse, men may develop greater psychological and physiological connections than after engaging in other sexual behaviors. Such intimate experiences not only foster trust in sexual partners but may also make one-night stands more psychologically acceptable and common. Anal intercourse can also reflect an individual's sexual exploration and adventurous spirit. MSM who have engaged in anal intercourse may gain a deeper understanding of their sexual identity and emotional needs, making them more open to casual, non-committal, emotional, and sexual relationships. However, anal intercourse is a high-risk behavior for STIs such as HIV and syphilis, and an increase in the number of sexual partners combined with a lack of effective safety measures may elevate the risk of contracting STIs. Therefore, enhancing health education and promoting safe sexual practices within MSM communities is crucial. The close relationship between anal intercourse and one-night stands reflects complex emotional and psychological dynamics and serves as a reminder that attention to health risks is paramount while enjoying sexual freedom. It is recommended to develop a digital warning system that triggers risk assessments for users frequently searching for “bareback anal intercourse” on dating apps as well as to provide information on HIV testing locations, thereby increasing the awareness of infection risks.

### 4.5 Alcohol, drug use, and aphrodisiacs

Recent studies have drawn considerable attention to the impact of alcohol consumption on MSM sexual behavior (32, 33). The findings indicate that MSM who engage in sexual activities with homosexual partners under the influence of alcohol, drugs, or performance-enhancing substances are 389% more likely to experience one-night stands.

Alcohol and drug use often diminish an individual's self-control and judgment, making them more susceptible to decisions that they would not typically make. Therefore, MSM may be more inclined to seek immediate sexual gratification without considering the associated health risks and emotional burdens. This behavior often arises from instant hedonism rather than from long-term considerations. Many MSM integrate drinking and drug use into social situations to seek acceptance and affirmation. In an enjoyable and relaxed atmosphere, sexual behavior is often viewed as a means of release and self-exploration, thereby increasing the incidence of one-night stands. Cultural factors should not be overlooked. In certain cultures or communities, the sexual behavior of MSM is regarded as a social activity, with alcohol and drugs seen as catalysts for facilitating such interactions. However, the health risks associated with this behavior indicate the necessity for enhanced education and preventive measures directed toward this population. Promoting awareness of safe sexual practices and encouraging healthy lifestyles are essential strategies to mitigate this phenomenon. By reducing or avoiding behaviors linked to alcohol consumption and drug use, including performance-enhancing substances, individuals can minimize potential health risks.

### 4.6 Belief in condom efficacy

The use of condoms is widely recognized as a critical means of preventing HIV and other STIs (34, 35). The findings of this study indicated that participants who believe in the efficacy of condoms for HIV prevention during homosexual encounters are 195% more likely to engage in one-night stands.

Furthermore, the willingness to use condoms and the habits surrounding their use are closely associated with sexual attitudes. Individuals who hold positive views about condoms may be more proactive in exploring their emotional and sexual behaviors; viewing one-night stands as an acceptable option. This phenomenon is particularly pronounced in certain communities where sexual behavior is normalized and condom use is expected. In some social circles, one-night stands are perceived as a form of relaxation and self-expression; thus, even those who understand the risks of HIV may participate because of social pressures or situational influences. While the effectiveness of condoms is well-established, it underscores the need to stress the importance of consistent condom use as a protective measure in sexual education and risk interventions (36). Through a comprehensive educational and support system, we can foster a deeper awareness of safety and effectively reduce the incidence of one-night stands among MSM and the associated health risks. Therefore, it is crucial to promote best practices for safe behavior while simultaneously offering multiple protective options, including pre-exposure prophylaxis (PrEP) and post-exposure prophylaxis (PEP), to ensure health and safety (37).

### 4.7 Regular homosexual partners

Stable sexual partnerships offer emotional and security support. Stable sexual partnerships typically indicate emotional attachment and trust, which facilitate smooth communication between the partners. This fosters a deeper emotional connection and predisposes both parties to choose one another in sexual encounters, diminishing their motivation to pursue short-term relationships. The findings of this study suggest that men with stable homosexual partners are 58% less likely to engage in one-night stands.

Interactions between stable partners are more likely to be grounded in mutual understanding and resonance rather than solely in physical needs. In such situations, MSM are more inclined to remain loyal to their partners and to avoid sexual relationships with others. This loyalty manifests not only physically but is also closely linked to the emotional commitment of both parties, thereby reducing the incidence of one-night stands. However, stable partnerships do not guarantee absolute safety. Factors such as partners' sexual health status, communication quality, and the external social environment may influence the health and safety of stable relationships. Understanding these dynamics is crucial for public health policymakers to develop effective preventive measures and intervention strategies. Encouraging and promoting the formation of stable partnerships, along with providing education on sexual health and emotional support, can significantly reduce the incidence of one-night stands among MSM, thereby mitigating the associated health risks.

### 4.8 Knowledge of partner's HIV status

Understanding a partner's HIV status not only enhances an individual's sense of safety but also increases sensitivity to the risks associated with sexual behavior. The findings of this study indicated that participants who knew their partners' HIV status were 38% less likely to engage in casual sex. This underscores the significance of the right to know and the vital role of information in sexual behavior.

When MSM are aware of their partners' health status, they are better equipped to assess risks and make informed decisions. This transparency reduces uncertainty and encourages more cautious behavioral choices, thereby decreasing the prevalence of casual sex. Furthermore, the awareness of a partner's HIV status often fosters open communication and health education. When partners can openly discuss their health status, both parties are more likely to take preventive measures, such as using condoms, which further mitigates the risk. This effective communication not only enhances awareness of HIV and other sexually transmitted diseases but also promotes safe practices in sexual behavior. However, it is important to note that this phenomenon does not imply that all individuals will completely avoid casual encounters simply because they understand their partners' health status. In some instances, individuals may still seek short-term sexual relationships because of environmental factors, social pressures, or personal preferences even when they are aware of their partners' status. Thus, educational and intervention strategies targeting male populations should not only emphasize the importance of the right to know but also consider individual motivations and social pressures to effectively reduce the risk of STIs. Understanding the homosexual partners' HIV status is crucial for lowering the incidence of casual sex, and this awareness undoubtedly supports efforts to promote sexual education and safe sexual behaviors. It is recommended to strengthen testing and disclosure policies, promote the concept of Undetectable = Untransmittable (U = U), and enhance partner notification systems, which are essential strategies for reducing the risk of STIs (38, 39).

The limitations of this study can be attributed to several factors. First, as this was a cross-sectional study, the self-report provided by the participants may have been biased. Due to the sensitivity surrounding casual sex, participants may experience social desirability effects that could lead them to conceal their true experiences during the survey. This inaccuracy in self-reporting can result in data bias, thereby affecting the evaluation of associated factors. Additionally, the representativeness of the study sample may be limited, as the participants may not adequately reflect the overall characteristics of MSM. The sample was recruited online and did not include MSM from fixed locations, which potentially restricts the generalizability and applicability of the research findings. Furthermore, the survey process may not have sufficiently considered the multiple intersecting factors influencing casual sex behaviors among MSM, such as cultural background, economic status, and individual psychological conditions. Variations in social contexts and individual differences may lead to diverse behavioral patterns, making it challenging to fully understand the complexity of the influencing factors through a one-dimensional analysis. Notably, while we measured self-reported alcohol and drug use during sex, we did not specifically query the use of illegal drugs commonly associated with high-risk sexual encounters among MSM (e.g., methamphetamine, mephedrone) nor the phenomenon of chemsex, which involves prolonged and often group sexual sessions under the influence of such substances. The conflation of all substance use into a single binary variable may underestimate the specific risks posed by chemsex-related one-night stands. Moreover, the absence of questions regarding certain preventive medications (such as PrEP) during one-night stands represents a missed opportunity to contextualize our findings within the current biomedical prevention landscape. Given that PrEP use is increasing among Chinese MSM and may alter risk perceptions and condom negotiation dynamics during casual encounters, future research should integrate chemsex and PrEP cascade indicators to inform more precise, combination HIV prevention approaches tailored to Internet-using MSM. Therefore, future multi-center prospective cohort follow-up studies should be conducted to validate the findings of this study.

## 5 Conclusions

This cross-sectional study investigated the prevalence and determinants of one-night stands among Internet-recruited MSM in China, with the aim of generating evidence to inform targeted HIV prevention interventions for this digitally connected subpopulation. Our findings revealed that one-night stands were highly prevalent (44.7%) in this sample, and were independently associated with a distinct risk profile encompassing sociodemographic characteristics (older age), partner-seeking channels (both offline fixed venues and online dating apps), sexual practices (anal intercourse), and situational factors (substance use during sex). Notably, while belief in condom efficacy increased the likelihood of one-night stands—suggesting a risk compensation effect—having a regular partner and knowing a partner's HIV status served as significant protective factors. For clinicians and peer educators, the heightened risk among MSM who combine substance use with sex highlights the urgent need to incorporate substance use screening and harm reduction counseling into routine HIV testing services. For policymakers, the protective effects of regular partnerships and HIV status disclosure support continued investment in stigma reduction, U=U awareness campaigns, and scalable HIV self-testing programs to facilitate serostatus communication. Addressing the complex interplay of digital environments, situational risks, and relationship dynamics identified in this study will be essential to a more tailored, effective HIV prevention landscape for Chinese MSM.

## Data Availability

The raw data supporting the conclusions of this article will be made available by the authors, without undue reservation.
